# Human metapnuemovirus infections in hospitalized children and comparison with other respiratory viruses. 2005-2014 prospective study

**DOI:** 10.1371/journal.pone.0173504

**Published:** 2017-03-16

**Authors:** María Luz García-García, Cristina Calvo, Cristina Rey, Beatriz Díaz, Maria del Mar Molinero, Francisco Pozo, Inmaculada Casas

**Affiliations:** 1 Pediatrics Department, Severo Ochoa Hospital, Leganés, Madrid, Spain; 2 University Alfonso X el Sabio, Madrid, Spain; 3 Traslational Research Network in Pediatric Infectious Diseases (RITIP), Madrid, Spain; 4 TEDDY Network Member (European Network of Excellence for Pediatric Clinical Research, Bari, Italy); 5 Respiratory Viruses and Influenza Unit, National Microbiology Center (ISCIII), Madrid, Spain; Kliniken der Stadt Köln gGmbH, GERMANY

## Abstract

**Background:**

Human metapneumovirus (HMPV) has an important etiological role in acute lower respiratory infections in children under five years. Our objectives were to estimate the relative contribution of HMPV to hospitalization in children with acute respiratory infection, to define the clinical and epidemiological features of HMPV single and multiple infections, and to compare HMPV infections with respiratory syncytial virus (HRSV), rhinovirus (HRV), adenovirus and human bocavirus infections in the same population.

**Methods and findings:**

A prospective study performed on all children less than 14 years of age with a respiratory tract disease admitted to a secondary hospital between September 2005- June 2014. Clinical characteristics of patients were analyzed. Nasopharyngeal aspirate was taken at admission for viral study with polymerase chain reaction for 16 respiratory viruses.

A total of 3,906 children were included. At least one respiratory virus was detected in 75.2% of them. The most common identified virus was HRSV, followed by HRV. HMPV was detected in 214 cases (5.5%); 133 (62%) were single infections and the remaining were detected in coinfection with other viruses. 90.7% cases were detected between February and May. Children’s mean age was 13.83 ± 18 months. Fever was frequent (69%), and bronchiolitis (27%), and recurrent wheezing (63%) were the main clinical diagnosis. Hypoxia was present in 65% of the patients and 47% of them had an infiltrate in X-ray. Only 6 (2.8%) children were admitted to the intensive care unit. Only the duration of the hospitalization was different, being longer in the coinfections group (p <0.05). There were many differences in seasonality and clinical characteristics between HMPV and other respiratory viruses being more similar to HRSV.

**Conclusions:**

HMPV infections accounted for 5.5% of total viral infections in hospitalized children. The clinical characteristics were similar to HRSV infections, but seasonality and clinical data were different from other viral infections.

## Introduction

Human metapneumovirus (HMPV), described in the Netherlands in 2001 [1] is an RNA virus belonging to the Pneumoviridae family, genus *Metapneumovirus* [2]. Two main genetic lineages A and B have been identified to date. The phylogenetic studies showed a high similarity to the respiratory syncytial virus (HRSV), with which it shares morphological and disease spectrum similarities [3]. Upper and lower respiratory tract infections from common colds to pneumonia have been attributed to HMPV, with bronchiolitis being one of the main clinical signs of primary infection in hospitalized patients [4]. A recent meta-analysis has provided evidence that HMPV has an important etiological role in acute lower respiratory infections in children less than five years [5].

Our objectives were to estimate the relative contribution of HMPV to hospitalization in children with acute respiratory tract infection in Spain and to define the clinical and epidemiological features of HMPV single and multiple infections. Also we compared HMPV infections with HRSV infections and with other common respiratory viruses over an extended period.

## Patients and methods

### Ethics statement

The study was approved by The Medical Ethics Committee of the Instituto de Salud Carlos III. Informed written consent was obtained from parents or legal guardians.

### Clinical assessment

The study population comprised all children between the first month of life and 14 years of age with a respiratory tract disease admitted to the secondary public hospital Severo Ochoa (Leganés, Madrid), between September 2005 and June 2014 which corresponded to nine consecutive seasons. All patients were evaluated by an attending physician. Clinical characteristics of patients were analyzed. During the hospital stay, and as part of the study, a physician filled out a study questionnaire with the clinical data.

Upper respiratory tract infection (URTI) was diagnosed in patients with rhinorrhea and/or cough and no signs of wheezing, dyspnea, crackles or bronchodilator use, with or without fever. Acute expiratory wheezing was considered to be bronchiolitis when it occurred for the first time in children aged less than 2 years following the McConnockie classical criteria [6]. All other episodes of acute expiratory wheezing were considered to be recurrent wheezing [7]. Asthma was diagnosed by the National Asthma Education and Prevention Program guidelines [8]. Laryngotracheobronchitis was associated with inspiratory dyspnea and wheezing. Laryngitis was related to inspiratory dyspnea without wheezing. Cases with both focal infiltrates and consolidation in chest X-rays were, in the absence of wheezing, classified as pneumonia.

### Virus detection

Clinical specimens consisted of a nasopharyngeal aspirate (NPA) taken from each patient at admission. All clinical specimens were sent for virological investigation to the Respiratory Virus and Influenza Unit at the National Microbiology Center (ISCIII, Madrid, Spain), NPAs were processed within 24 hours after collection. Upon receipt, three aliquots were prepared and stored at -70°C.

RNA and DNA from 200 μl-aliquots of NPA were extracted by using the QIAamp Mini Elute Virus spin kit in an automated extractor (QIAcube, Qiagen, Valencia, CA). From 2005 to 2010, three conventional multiplex RT-nested-PCR assays were performed to detect a total of sixteen respiratory viruses [9,10,11]. From 2011 to 2014, detection of HMPV and the other respiratory virus were performed by real time multiplex RT-PCR assays, not published yet, but based on the same equivalent conventional methods (9, 10, 11).

### Statistical analysis

Values were expressed as percentages for discrete variables, or as mean and standard deviation for continuous variables. Clinical characteristics of patients with single infections associated to HMPV were compared with those associated with coinfections of HMPV with other respiratory viruses. Single HMPV infections were also compared with single infections by HRSV, human rhinovirus (HRV), adenovirus (HAdV) and human bocavirus (HBoV). Clinical characteristics and laboratory variables were compared using the Student t-test, the Mann-Whitney U test, the chi-2 test, and Fisher’s exact test. A two-sided value of P < 0.05 was considered statistically significant. Results were adjusted for age. All analyses were performed using the Statistical Package for the Social Sciences (SPSS), Version 21.0.

## Results

A total of 3906 children under 14 years of age, admitted to the Severo Ochoa Hospital with acute respiratory tract infections were included. The mean age was 21.68 ± 33.8 months. At least one respiratory virus was detected in 75.2% of them. The most common identified virus was HRSV, followed by HRV, and HAdV (Table 1). HMPV was detected in 214 cases (5.5%).

**Table 1:** Total identified viruses (simple detections and coinfections).

Virus	N = 3906 patients
HRSV	1202 (30.8%)
Rhinovirus	1175 (30.4%)
Adenovirus	805 (20.6%)
HMPV	214 (5.5%)
Influenza	182 (4.7%)
PIV	240 (6.1%)
HBoV	334 (8.6%)
EV	85 (2.2%)
CoV	82 (2.1%)
Coinfections	887 (22.7%)

HRSV: respiratory syncytial virus, HMPV: human metapneumovirus, PIV: parainfluenza virus, HBoV: human bocavirus, EV: enterovirus, CoV: coronavirus.

### Clinical data of HMPV infections

**Fig 1:**
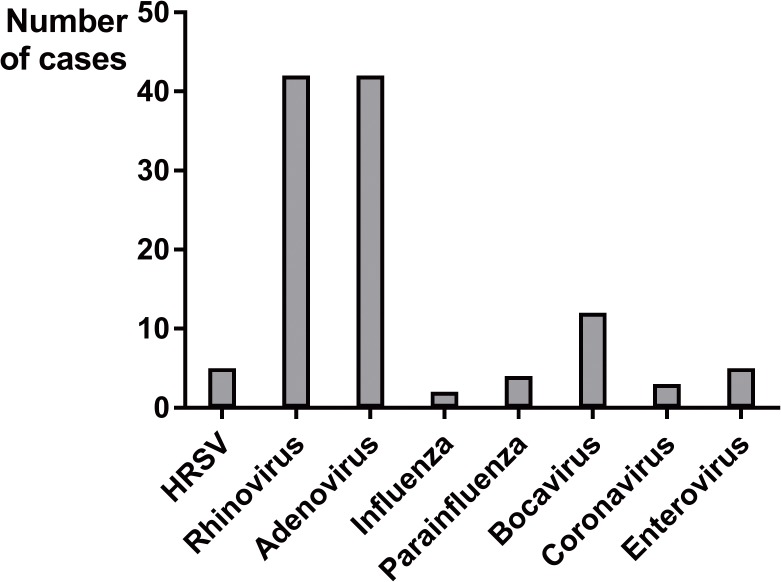
Multiple infections detected with HMPV.

Out of 214 children with an infection associated to HMPV; 133 (62%) were single infections, and the remaining 81 were detected in coinfection with other respiratory viruses (38%). The most frequent coinfections were with adenovirus and rhinovirus (Fig 1).

**Fig 2:**
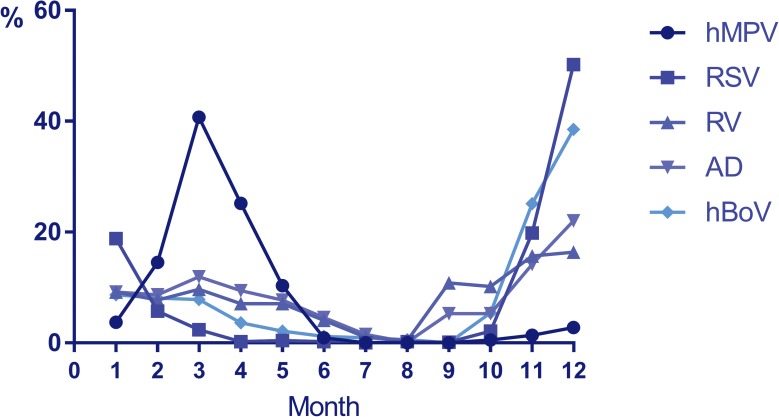
Monthly distribution of studied viruses (monthly percentage of each virus).

Monthly distribution of HMPV infections is shown in Fig 2, being 90.7% between February and May.

Clinical data of HMPV infections are shown in Table 2. Children’s mean age was 13.83 ± 18 months, 58% (125/214) were males, and 15.5% were preterm infants. Fever was frequent (69%; 148/214), and bronchiolitis (27%; 47/214), and recurrent wheezing (63%; 108/214) were the main clinical diagnosis. Hypoxia was present in 65% (140/214) of the patients; with a mean duration of 2.9 ± 2 days and 47% (82/214) of them had an infiltrate in X-ray. The length of the stay was 4.4 ± 2.3 days. Only 6 (2.8%) children were admitted to the intensive care unit (ICU). Infants less than 6 months of age have less proportion and duration of fever, but longer duration of hypoxia and stay. Diagnosis of bronchiolitis was more frequent in this group (Table 3). Single infections were compared with coinfections of HMPV with other viruses (Table 2), and only duration of the hospitalization was different, being longer in the coinfections group (p <0.05).

**Table 2:** Clinical data and comparison of single HMPV infections vs coinfections.

	HMPV single (N = 133)	HMPV coinfection (N = 81)	P	OR (IC 95%)
Sex: males	77 (57.9%)	48 (59.3%)	0.844	1.036 (0.73–1.470)
Age ± SD (months)	14.37 ± 20.85	12.9 ± 11.69	0.606	
Fever ≥ 38°	90 (67.7%)	58 (71.6%)	0.545	1.125 (0.764–1.655)
Maximum Tª (°C)	38.83 ± 0.62	38.84 ± 0.62	0.900	
Days of fever	2.6 ± 1.6	2.9 ± 1.7	0.381	
SatO2 < 95%	83 (62.4%)	57 (70.4%)	0.235	1.255 (0.854–1.845)
Days of hypoxia*	2.6 ± 1.8	3,2 ± 2.4	0.095	
Days of hospitalization*	4.2 ± 2.1	4.9 ± 2.6	0.042	
**X-ray**				
Infiltrate	43 (48.3%)	24 (46.2%)	0.804	
Normal	46 (51.7%)	28 (53.8%)		
**Diagnosis**			0.529	
Recurrent wheezing/ asthma	65 (60.7%)	43 (67.2%)		
Bronchiolitis	32 (29.9%)	15 (23.4%)		
Pneumonia	9 (8.4%)	4 (6.2%)		
Laryngitis	1 (0.9%)	2 (3.1%)		
**Prematurity**	20 (15.2%)	13 (16%)	0.860	1.043 (0.656–1.657)
**Antibiotic treatment**	34 (25.6%)	23 (28.4%)	0.650	1.092 (0.750–1.591)

*Mean ± standard deviation (SD)

**Table 3:** Clinical data of HMPV infections by age group.

	0–6 months N = 61	6–12 months N = 77	1–2 years N = 50	>2 years N = 26	p
Fever	28 (46%)	56 (73%)	42 (84%)	22 (88%)	0.0001
Days of fever (SD)	1.9 (1.2)	2.8 (1.5)	3.1 (1.7)*	2.9 (2.2)	0.014*
Hypoxia	33 (54%)	52 (67.5%)	37 (74%)	18 (72%)	0.126
Days of hypoxia (SD)	3.3 (2.2)	3.2 (2.3)	2.2 (1.2)*	2.5 (2)	0.016*
Days of stay (SD)	4.6 (2.7)	4.8 (2.4)	3.8 (1.3)*	4.7 (2.5)	0.047*
Infiltrate in X-ray	12 (29%)	31 (48%)	23 (53.5%)	16 (66%)	0.022
Bronchiolitis	41 (72%)	19 (25%)	2 (4%)	-	0.0001
ICU admission	2 (4.5%)	0	2 (5%)	1 (5%)	0.355

SD: standard deviation, ICU: intensive care unit.

* Group age with significant differences.

### Comparison of HMPV infections with other respiratory viruses

Single HMPV infections (n = 133) were selected and compared with single infections of the most frequent viruses detected in the same period; HRSV (n = 766), rhinovirus (n = 651), adenovirus (n = 355) and HBoV (n = 84).

#### HMPV vs HRSV

Clinical data of single infections associated to HRSV and the comparison with HMPV single infections are shown in Table 4. HRSV was detected in younger infants (p = 0.002), who had hypoxia (p = 0.04) and bronchiolitis diagnosed more frequently than HMPV group (p < 0.001). Recurrent wheezing (p< 0.001) and antibiotic treatment (p = 0.05) were more frequent in HMPV group.

**Table 4:** Clinical data and comparison between single HMPV and HRSV infections.

	HMPV N = 133	HRSV N = 766	P	OR (IC 95%)
Sex: male	77 (57.9%)	410 (53.5%)	0.35	1.163 (0.846–1.599)
Age ± SD (months)*	14.37 ± 20.85	9.67 ± 12.83	0.002	
Fever ≥ 38° (°C)	90 (67.7%)	488 (63.8%)	0.38	1.159 (0.828–1.623)
Máximum Tª*	38.83 ± 0.62	38.74 ± 0.62	0.23	
Days of fever*	2.6 ± 1.6	3,0 ± 2.7	0.24	
SatO2 < 95%	83 (62.4%)	544 (71.1%)	0.044	0.717 (0.521–0.989)
Days of hypoxia*	2.6 ± 1.8	3.0 ± 2.2	0.14	
Days of hospitalization*	4.2 ± 2.1	4.6 ± 2.5	0.05	
**X-ray:**				
Infiltrate	43 (48.3%)	259 (49.9%)	0.7	0.947 (0.645–1.391)
Normal	46 (51.7%)	260 (50.1%)		
Leukocytes/mm3 *	11732± 4446	12461 ± 12500	0.46	
C-reactive protein (mg/dL)*	34 ± 38	28 ± 40	0.24	
**Diagnosis**:			<0.001	
Recurrent wheezing /asthma	65 (60.7%)	228 (30.5%)		
Bronchiolitis	30 (29.9%)	485 (65%)		
Pneumonia	9 (8.4%)	26 (3.5%)		
Laryngitis	1 (0.9%)	8 (1.1%)		
Prematurity	20 (15,2%)	99 (13%)	0.5	1.160 (0.751–1.792)
Antibiotic treatment	34 (25.6%)	138 (18%)	0.05	1.452 (1.021–2.065)
ICU admission	4 (2.2%)	22 (2.87%)	0.77	

*Mean ± standard deviation (SD)

HMPV: human metapneumovirus, HRSV: respiratory syncytial virus.

#### HMPV vs HRV

Clinical data of single HRV infections and the comparison with HMPV single ones are shown in Table 5. Children with HMPV infections are younger than HRV group (p< 0.001), and had more frequent fever (p<0.001) and hypoxia (p = 0.02); longer duration of hypoxia (p = 0.001) and hospitalization (p<0.001). A higher level of C-reactive protein was found in HRV group (p = 0.009).

**Table 5:** Clinical data and comparison between HMPV and rhinovirus single infections.

	HMPV N = 133	RHINOVIRUS N = 651	P	OR (IC 95%)
Sex: male	77 (57.9%)	398(61%)	0.357	0.856(0.615–1.192)
Age ± SD (months)*	14.37 ± 20.85	25.7±27.9	<0.001	
Fever ≥ 38°	90 (67.7%)	272(41.8%)	<0.001	2.386(1.674–3.385)
Maximum Tª	38.83 ± 0.62	38.7±0.6	0.08	
Days of fever*	2.6 ± 1.6	2.3±1.7	0.127	
SatO2 < 95%	83 (62.4%)	330(51%)	0.021	1.486(1.056–2.090)
Days of ipoxia*	2.6 ± 1.8	1.9±1.7	0.001	
Days of hospitalization*	4.2 ± 2.1	3.4±2.1	<0.001	
**X-ray:**				
Infiltrate	43 (48.3%)	178(43.1%)	0.440	1.152(0.805–1.648)
Normal	46 (51.7%)	235(57%)		
Leukocytes/mm3 *	11732± 4446	19609± 66000	0.35	
C-reactive protein (mg/dL)*	34 ± 38	53 ± 88	0.009	
**Diagnosis:**			0.30	
Recurrent wheezing/ asthma.	65 (60.7%)	530 (57.3%)		
Bronchiolitis	32 (29.9%)	253(27.4%)		
Pneumonia	9 (8.4%)	88(9.5%)		
Laryngitis	1 (0.9%)	14(2.4%)		
Prematurity	20 (15.2%)	80(12.4%)	0.191	1.341(0.871–2.066)
Antibiotic treatment	34 (25.6%)	141(21.7%)	0.321	1.209(0.833–1.754)
ICU admission	4 (2.2%)	20 (2.1%)	0.477	

*Mean ± standard deviation (SD)

HMPV: human metapneumovirus.

#### HMPV vs HAdV

Clinical data of single HAdV infections and the comparison with HMPV infections are shown in Table 6. Again, the HMPV group was a younger than the HAdV group (p <0.001), had more frequent hypoxia (p = 0.07) and longer duration of fever (p< 0.001). Prematurity was also more frequent (p = 0.06). HAdV group was diagnosed with pneumonia and laryngitis more frequently and with recurrent wheezing or asthma (p< 0.001) less commonly. A higher value of leucocytes (p = 0.001) and C-reactive protein (p = 0.59) were found in HAdV infected children.

**Table 6:** Clinical data and comparison of HMPV and adenovirus single infections.

	HMPV N = 133	ADENOVIRUS N = 335	P	OR (IC 95%)
Sex: male	77 (57.9%)	190(56.7%)	0.992	0.999(0.732–1.361)
Age ± SD (months)*	14.3±20.8	27.6±24.4	<0.001	
Fever ≥ 38°	90(67.7%)	221(66.6%)	0.984	1.003(0.725–1.389)
Maximum Tª	38.8±0.6	38.7±0.7	0.733	
Days of fever*	2.6 ± 1.6	3.6±2.8	0.001	
SatO2 < 95%	83 (62.4%)	176(53%)	0.07	1.335(0.970–1.837)
Days hypoxia*	2.6 ± 1.8	2.5±2.1	0.533	
Days of hospitalization*	4.2±2.1	4.1±2.4	0.596	
**X-ray:**				
Infiltrate	43 (48.3%)	131(53.5%)	0.313	0.841(0.600–1.178)
Normal	46 (51.7%)	114(46.5%)		
Leukocytes/mm^3^ *	11732± 4446	14361± 7440	0.001	
C-reactive protein (mg/dL)*	34 ± 38	49 ± 67	0.059	
**Diagnosis:**			<0.001	
Recurrent wheezing /Asthma	65 (60.7%)	162(48.5%)		
Bronchiolitis	32 (29.9%)	71(23%)		
Pneumonia	9 (8.4%)	55(16.5%)		
Laryngitis	1 (0.9%)	12(7.8%)		
Prematurity	20 (15.2%)	34(10.2%)	0.06	1.489(1.011–2.193)
Antibiotic treatment	34 (25.6%)	111(33%)	0.134	0.765(0.535–1.094)
ICU admission	4 (2.2%)	2 (1.2%)	0.335	

*Mean ± standard deviation (SD)

HMPV: human metapneumovirus, OR: odds ratio.

#### HMPV vs HBoV

Clinical data of single HBoV infections and the comparison with HMPV group are shown in Table 7. Children with HMPV infections were younger (p < 0.001) and bronchiolitis was more frequent (p = 0.002), with longer hospitalization (almost significant; p = 0.08). Pneumonia was more common in the HBoV group as well as the antibiotic treatment (p = 0.013). Leucocytes (p = 0.02) and C-reactive protein (p = 0.04) in blood were higher in HBoV infected children.

**Table 7:** Clinical data of single HBoV infections and comparison with HMPV.

	HMPV N = 133	HBoV N = 84	P	OR (IC 95%)
Sex: male	77 (57.9%)	55 (65.5%)	0.265	0.885 (0.718–1.093)
Age ± SD (months)*	14.37 ± 20.85	25.03 ± 23.87	0.001	
Fever ≥ 38°	90 (67.7%)	57 (67.9%)	0.977	0.997 (0.795–1.249)
Maximum Tª*	38.8 ± 0.6	38.9 ± 0.6	0.283	
Days of fever*	2.6 ± 1.6	2.9 ± 2.1	0.340	
SatO2 < 95%	83 (62.4%)	44 (52.4%)	0.144	1.176 (0.940–1.472)
Days of hypoxia*	2.6 ± 1.8	2.3 ± 1.5	0.237	
Days of hospitalization*	4.2 ± 2.1	3.7 ± 2.0	0.083	
**X-ray:**				
Infiltrate	43 (48.3%)	38 (62.3%)	0.091	0.796 (0.611–1.037)
Normal	46 (51.7%)	23 (37.7%)		
Leukocytes/mm^3^ *	11732± 4446	15603 ±7800	0.02	
C-reactive protein (mg/dL)*	34 ± 38	65 ± 78	0.04	
**Diagnosis:**			0.002	
Recurrent wheezing/ asthma	65 (60.7%)	48(58.5%)		
Bronchiolitis	32 (29.9%)	14 (17%)		
Pneumonia	9 (8.4%)	18 (22%)		
Laryngitis	1 (0.9%)	2 (2.4%)		
Prematurity	20 (15.2%)	8 (9.5%)	0.230	1.199 (0.922–1.558)
Antibiotic treatment	34 (25.6%)	35 (41.7%)	0.013	0.737 (0.565–0.960)
ICU admission	4 (2.2%)	1 (1.3%)	0.473	

*Mean ± standard deviation (SD)

HMPV: human metapneumovirus, HBoV: human bocavirus, OR: odds ratio.

### Viral seasonal and annual distribution

Monthly distribution of HMPV was significantly different (p< 0.001) from all other analyzed respiratory viruses. Comparison of monthly circulation is shown in Fig 2.

In relation to the percentage of annual infections, it was variable with a minimum of 2.3% of cases detected in 2006 and a maximum of 19.9% in 2009.

## Discussion

According to this large and both long series, i.e. nine consecutive epidemic seasons, of respiratory infections in hospitalized children, hMPV had an important role in infants and was associated with 5.5% of admissions. Up to 38% was detected in coinfection with other viruses, and had a typical seasonal distribution being mainly in spring. Recurrent wheezing was the most common clinical diagnosis, usually associated with fever and hypoxia. However, infants less than 6 months had less fever, and were usually diagnosed of bronchiolitis. Clinical and epidemiological data were significantly different between single hMPV infections and other respiratory viral infections.

The burden of HMPV infections in hospitalized children has been confirmed in other countries around the world [12]. In Jordanian children [13] of less than 2 years, hMPV was associated with 8.6% of cases, slightly higher than ours, probably because only young children were included. In Germany [14], 11.9% of hospitalized children with bronchitis, pneumonia or pharyngitis were positive for hMPV, but the study was not performed throughout the year, but only during the flu season (weeks 41 to 18). In Argentina [15], the proportion of HMPV infections in hospitalized children ranged from 18% in those less than 6 months of age and 5% in children under 5 years, which is very similar to our data. When children up to 15 year old were included in the U.S by Hahn et al [16], HMPV infections were 3% of respiratory infections in hospitalized children.

The proportion of coinfections with other respiratory viruses of up to 38% in our hospital, was higher than in three states of the U.S. [17] where they found 21% of multiple infections, but they did not test as many respiratory viruses as we did (only HRSV, influenza, and parainfluenza virus). Nevertheless, in Jordan, Schuster et al [13] found up to 53% of coinfections, and rhinovirus and adenovirus were frequently detected in coinfections, as in our country, but they also encountered a substantial number of coinfections with HRSV, being clearly higher than in Spain. This has also been described in other studies, such as Semple [18] in the United Kingdom, where coinfections between HMPV and HRSV were frequent and severe. However, in California [19], as in our study, only 1% of patients had coinfections between HMPV and HRSV. Probably, the seasonal circulation of HMPV is different among countries or geographical areas and allows that mixed infections were more or less frequent. Hence, in Jordan, HMPV was detected in winter and spring, and they were partly coincident with the circulation of HRSV. However, in Spain, circulation of both viruses was significantly different making coinfections unlikely. February, March, and April were the peak months of HMPV circulation in our country.

Clinical data associated with hospitalization due to HMPV was similar to other large studies. In a prospective, population-based surveillance study in the United States [17], with more than 600 cases, pneumonia (50%), bronchiolitis (22%) and asthma (14%) were the most common diagnosis. In our series, 47% of children had an infiltrate in X-ray, although most of them were diagnosed with recurrent wheezing based on our diagnostic criteria. Up to 53% of children in the US study, and 65% of our patients needed oxygen during the admission. In Jordanian children [13], clinical diagnoses were similar to ours, with bronchopneumonia and bronchiolitis being more frequent.

Prematurity is a well-known risk factor for hospitalization and severity in respiratory viral infections and has also been described in HMPV infections [16,20]. We also found an important proportion of infants with a history of prematurity (15%) in our series. No other significant proportion of underlying conditions were identified in our hospital, probably because it is a secondary center and the majority of attending children were previously healthy.

In our series, clinical differences have been found between HMPV infections and other respiratory viruses infections. The comparison with HRSV is relatively frequent in the literature. As in our results, clinical data of both virus infections are very similar. The global burden of HMPV infections is less than HRSV. In Guatemala [21], HMPV was less prevalent than HRSV (3% vs 41%) in hospitalized children, and HMPV infections were detected in older infants and with less severity. In Egypt[22] the proportion was 4% and 46%, clinical differences wer not found. In our study, the proportion was 5.5% for HMPV and 30.8% for HRSV. The older age of infants with HMPV is consistent in different studies [19,22,23] and also in our series. HMPV patients had less frequent hypoxia than HRSV ones, the duration of the hospitalization was shorter and the diagnosis of recurrent wheezing was more frequent. In addition, pneumonia was more common in the HMPV group.

Nevertheless, as far as we know, no other authors have specifically compared HMPV infections with other viruses. We compared single HMPV infections with single HRV, single HAdV, and single HBoV cases. The HMPV children were the youngest amongst all of them. Hypoxia and duration of the hospitalization are usually more frequent in HMPV group than in other respiratory viral infections, probably in relation to the lower age of the infants. Pneumonia, a higher rate of leukocytes and C-reactive protein were more frequent in HAdV or HBoV. Recurrent wheezing was more common in HMPV patients. Seasonality was also different because of the characteristic and singular circulation of HMPV with the highest incidence being in March and April.

In summary, HMPV infections accounted for 5.5% of total viral infections in hospitalized children. The clinical characteristics were similar to HRSV infections, but children were younger, and recurrent wheezing was more common. Seasonality and clinical data are different from other viral infections such as HRV, HAdV or HBoV that affect older children with a higher proportion of pneumonia. HMPV infections have a significant burden of disease, and the development of vaccines could prevent a substantial number of hospitalizations.

## Supporting information

S1 File Supporting information file. Fig A. Multiple infections detected with HMPV. Fig B. Monthly distribution of studied viruses (monthly percentage of each virus).
(XLSX)
